# The hemochromatosis protein HFE 20 years later: An emerging role in antigen presentation and in the immune system

**DOI:** 10.1002/iid3.158

**Published:** 2017-04-19

**Authors:** Alexandre Reuben, Jacqueline W. Chung, Réjean Lapointe, Manuela M. Santos

**Affiliations:** ^1^ Centre de Recherche du Centre Hospitalier de l'Université de Montréal (CRCHUM) Montréal, Québec Canada; ^2^ Département de Médicine Université de Montréal Montréal, Québec Canada; ^3^ Institut du Cancer de Montréal Montréal, Québec Canada

**Keywords:** Antigen processing and presentation, hereditary hemochromatosis, HFE, MHC

## Abstract

**Introduction:**

Since its discovery, the hemochromatosis protein HFE has been primarily defined by its role in iron metabolism and homeostasis, and its involvement in the genetic disease termed hereditary hemochromatosis (HH). While HH patients are typically afflicted by dysregulated iron levels, many are also affected by several immune defects and increased incidence of autoimmune diseases that have thereby implicated HFE in the immune response. Growing evidence has supported an immunological role for HFE with recent studies describing HFE specifically as it relates to MHC I antigen presentation.

**Methods/Results:**

Here, we present a comprehensive overview of the relationship between iron metabolism, HFE, and the immune system to better understand the origin and cause of immune defects in HH patients. We further describe the role of HFE in MHC I antigen presentation and its potential to impair autoimmune responses in homeostatic conditions, a mechanism which may be exploited by tumors to evade immune surveillance.

**Conclusion:**

Overall, this increased understanding of the role of HFE in the immune response sets the stage for better treatment and management of HH and other iron‐related diseases, as well as of the immune defects related to this condition.

## Introduction

The human body employs multiple mechanisms in order to maintain metabolic homeostasis. To maintain this balance, the immune system is of paramount importance, providing protection against pathogens such as bacteria, fungi, parasites, and viruses, in addition to guarding against malignant transformations and cancer development 1. However, several key metabolic elements may be co‐opted by pathogens attempting to infiltrate and colonize the host 2 in which metabolic pathways are targeted for pathogen proliferation and persistence 3. This presents an intricate undertaking in which the immune system must also balance between attacking foreign bodies and sparing host cells to prevent the risk of developing autoimmune diseases 4. Thus, the metabolic and immune systems are tightly regulated to benefit the host.

Links have been established between metabolism and immunity with discoveries revealing the impact of glycolysis on T cell maturation and activation 5. Furthermore, proteins involved in iron homeostasis may impact lymphocyte populations that can lead to abnormal ratios of T cell subsets 6, 7. In particular, the hemochromatosis (HFE) protein is at the interface of iron metabolism and immunity. HFE acts as an iron sensor for the body and regulates iron absorption in the small intestine and iron recycling by macrophages 8. When mutated, HFE is associated with the development of hereditary hemochromatosis (HH), a disease characterized by excess iron in the body 9. Here, we review an increasing number of studies that provide evidence of a direct link between HFE and the immune system, most notably linking HFE to antigen presentation by major histocompatibility complex class I (MHC I) molecules.

## HFE: Discovery and Iron‐Related Function

HFE was identified in 1996 as the gene responsible for HH 9. HH is an autosomal‐recessive disorder characterized by the overabsorption of iron in the intestine and the storage of excess iron in essential organs, such as the heart, liver, and pancreas, which can lead to their irreversible destruction 10. More recently, HH has been attributed to the complete or partial loss of hepcidin, a hormone produced by the liver, resulting in heightened iron entry into the bloodstream 11. Most hereditary cases of hemochromatosis in humans arise from genetic mutations within components of the iron‐sensing machinery that regulates hepcidin. Transcription of hepcidin is dependent upon iron signaling through these components, assembled as a membrane‐associated signaling complex and consisting of bone morphogenetic proteins (BMPs), BMP receptors, hemojuvelin (HJV), and other proteins that include transferrin receptor 1 (TfR1) and HFE 11.

**Figure 1:**
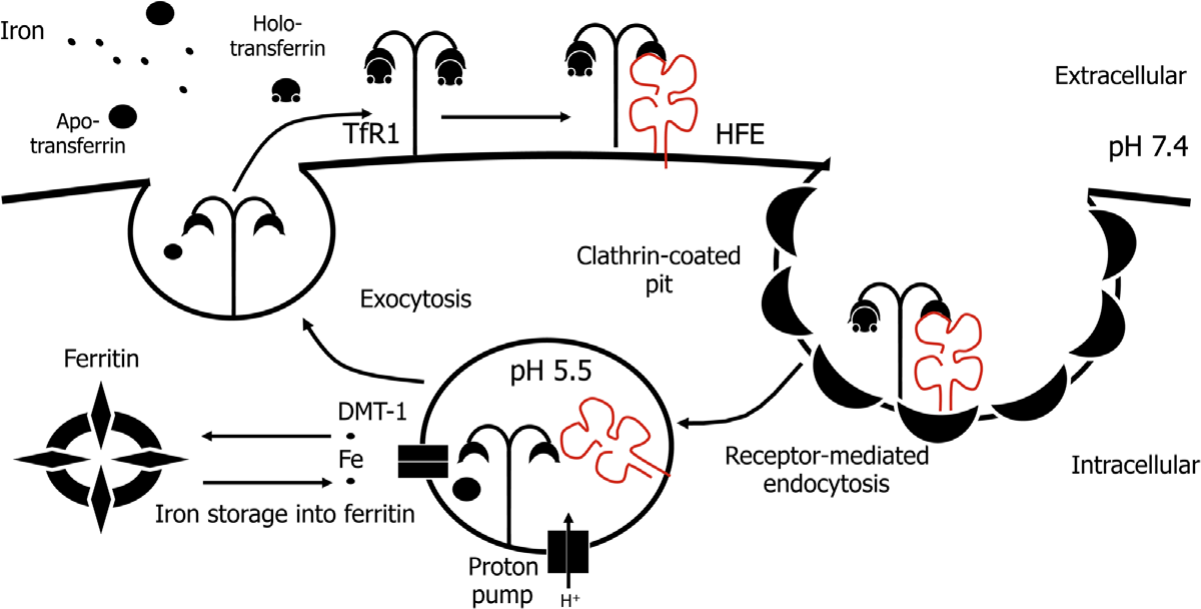
HFE as an iron sensor and the TfR1/Tf cycle for cellular iron uptake. The TfR1 is located at the cell surface where it binds Tf‐bound iron. HFE is also located at the cell surface and competes with Tf for binding to TfR1. The TfR1/Tf or TFR1/HFE complex is internalized by clathrin‐dependent endocytosis before iron is released from the TfR1/HFE complex in endosomes acidified to a pH of 5.5. Iron is then exported from the endosomes to the cytosol through the DMT‐1 transporter and is typically stored as ferritin in the cytosol.

Initial studies on the function of HFE revealed that it is involved in modulating iron uptake by the transferrin receptor 1 (TfR1) 12, 13, 14 (recently reviewed in 15) (Fig. 1). At the cell surface, TfR1 binds to transferrin (Tf), a plasma molecule that binds circulating iron, and forms a complex which is then endocytosed. Iron is released from the TfR1‐Tf complex through endosomal acidification and is exported to the cytosol by way of the divalent metal transporter 1 (DMT1) 16. Iron can then be used for metabolic purposes or stored within ferritin, the major iron storage protein, and the apo‐TfR1‐Tf complex is recycled to the surface, completing the so‐called transferrin cycle for cellular iron uptake 17. HFE expressed at the cell surface competes with Tf for binding to TfR1, reducing TfR1‐Tf interactions, and negatively regulating iron uptake 18. As an iron metabolism “sensor” 19, HFE regulates the downstream production of hepcidin, the major systemic regulatory hormone of iron metabolism 20. Iron‐sensing involving HFE and TfR1 triggers a signaling cascade through the BMP/SMAD pathway to induce hepcidin transcription 11. Hepcidin mediates iron absorption and distribution primarily by blocking iron efflux from cells. The iron exporter, ferroportin, acts as a receptor for hepcidin and is present on macrophages, hepatocytes, and the basolateral surface of enterocytes; binding to hepcidin results in the internalization and degradation of ferroportin, thereby inhibiting iron exit from cells 21, 22. In enterocytes, diminished iron efflux via hepcidin results in limiting iron uptake and inhibiting intestinal iron absorption 23, while ferroportin degradation by hepcidin in macrophages prevents iron recycling and leads to the intracellular accumulation of iron 24. When these responses are sustained for long periods, as during chronic infections and autoimmune diseases, they can lead to the development of anemia of chronic disease (ACD) 25. This condition is, therefore, associated with iron‐restricted erythropoiesis, because despite the presence of adequate iron in the body, it remains inaccessible to meet erythropoietic demands.

Hereditary forms of hemochromatosis arising from hepcidin deficiency are primarily related to the C282Y mutation in HFE, with rare forms involving mutations in HJV and hepcidin 26. HH in association with HFE_C282Y_ is most common among Caucasians, with a relatively high prevalence of 1 in 10 within this population 26. Although C282Y homozygosity predisposes individuals to iron‐loading, the mutation has low penetrance with only a small percentage of patients significantly impacted with disease progression 11, 26, 27. HFE‐related hemochromatosis (HH type 1) is often dependent upon concomitant conditions and additional factors that modulate the expressivity and disease developing into organ damage 26. Alcohol abuse and genetic modifiers such as polymorphisms in genes involved in hepcidin/ferroportin regulation or in antioxidant defense and tissue repair, have been identified as having impact on the phenotype of HFE‐related hemochromatosis 11.

While there are no doubts that HFE plays an important role in iron metabolism, its remarkable similarity to the structure of MHC I molecules raises questions on HFE involvement in the immune response, specifically through antigen presentation.

## Antigen Presentation by MHC I Molecules

MHC molecules are host‐cell glycoproteins at the cell surface that are specialized in presenting antigens to T lymphocytes. Antigen presentation is part of an active monitoring mechanism to detect harmful or invading agents, and involves generating peptides (antigens) from endogenous or exogenous proteins for display by MHC I or MHC II molecules, respectively. MHC I is present in all nucleated cells in the body, whereas MHC II tends to be limited to professional antigen presenting cells (pAPC) such as macrophages, dendritic cells (DC), and B lymphocytes. The display of MHC‐bound peptides to T lymphocytes initiates an immune response for the effective elimination of infected and damaged cells while avoiding autoimmunity by discriminating between “foreign” and “self” antigens. Therefore, a perfect balance must be achieved between the specific and promiscuous binding of antigens that can be presented on the same MHC molecules 28.

**Figure 2:**
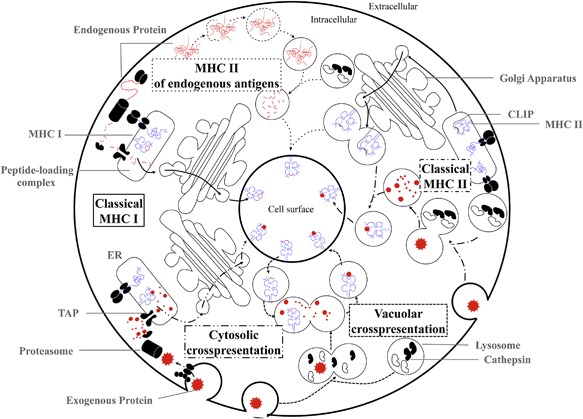
MHC I and II antigen presentation pathways. Schema depicting overviews of the classical and cross‐presentation pathways through MHC I and II.

Classical MHC I molecules display peptides derived from antigens within the cell, including those that are from “self,” pathogen‐associated, or cancer‐associated proteins. Prior to their presentation, these proteins are digested in the cytosol and are transported into the endoplasmic reticulum (ER) by the transporter associated with antigen processing (TAP), which forms a peptide‐loading complex (PLC) with nascent MHC I molecules stabilized by the chaperones tapasin, calreticulin, and ERp57 (Fig. 2) 29. The PLC ensures that MHC I molecules are properly bound to peptides for surface transport and presentation; peptides are selected based on their high affinity for MHC I molecules and ability to confer MHC I stability 30. In contrast, MHC II antigen presentation involves peptides derived from proteins taken up from the extracellular space. Initially, exogenous proteins are digested within endocytic vesicles through the protease activity of cathepsin S (CatS) in lysosomes 31. Endolysosomes then fuse with vesicles containing MHC II molecules that are associated with the invariant chain peptide CLIP. Human leukocyte antigen (HLA)‐DM is also present in these vesicles and catalyzes the displacement of CLIP with the generated peptides, permitting transport of peptide‐loaded MHC II molecules to the cell surface (Fig. 2) 32.

**Figure 3:**
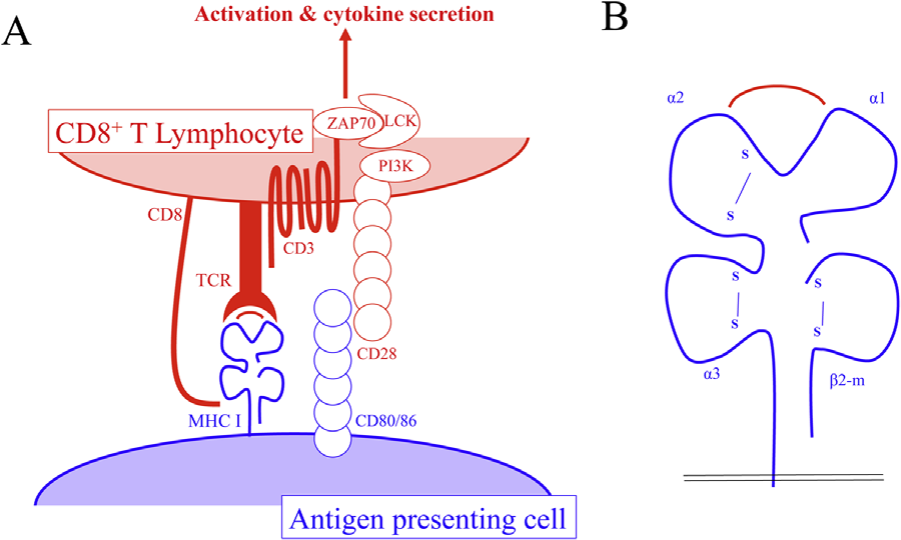
The immunological synapse (IS) and MHC I molecule. A) Interface between APC and CD8^+^ T lymphocytes. The centre of the IS consists of the MHC I molecule presenting a peptide, the TCR, and CD8 co‐receptor, which binds the MHC I molecule/peptide complex. B) Magnification of the MHC I molecule with bound peptide. The MHC I molecule structure consists of a heavy chain of three alpha domains (α1–3) anchored by a transmembrane domain and a β2‐microglobulin (β2‐m) light chain.

Antigen presentation is without consequence if the presented antigens are not recognized by the T cell receptor (TCR) on T lymphocytes 33. MHC I/peptide complexes are recognized exclusively by cytotoxic T lymphocytes (CTL) that also express the CD8 co‐receptor, whereas MHC II/peptide complexes interact with helper T cells expressing the CD4 co‐receptor. T lymphocytes bind to the MHC/peptide complexes, forming an immunological synapse (IS) that includes the TCR and CD3 co‐signaling molecule, along with other co‐stimulatory molecules 34. For MHC I, the IS involves the CD8 co‐receptor at the interface between the APC and T cell (Fig. 3A). The MHC I structure consists of a heavy α chain that presents peptides within a groove created by the α1‐2 domains (Fig. 3B). The TCR binds the peptide and polymorphic residues within the α1‐2 domains while CD8 binds the α3 domain of the MHC I (Fig. 3A and B) 35. This interaction engages the CD3 molecule present at the IS and leads to subsequent signaling 36 which results in the following: cytokine production, activation of CD8^+^ T lymphocytes, and lysis of infected cells through production of granzyme and perforin 37, 38 or FAS‐ligand binding 39, which leads to apoptosis of infected cells.

## Regulation of MHC I Antigen Presentation

In response to inflammatory stimuli, the immune system tightly regulates MHC I antigen processing to maintain tolerance to self‐antigens and tend to the immediate needs of the host. The promoter region of MHC I genes are activated by multiple pathways that enable dynamic expression under such different conditions 40, permitting constant immune surveillance, constitutive expression within tissue sites, and immediate response to harmful agents or pathogens 40. Cytokines, hormones, and certain chemicals modulate MHC I expression 41. IL‐2, IFN‐γ, and GM‐CSF are the major cytokines deployed during the immune response to pathogens and induce an increase in MHC I expression and efficiency of MHC I processing 41. In particular, IFN‐γ is responsible for the upregulation of MHC I expression to enhance the CTL response 42 and can induce components of the antigen processing pathway such as the proteasome subunits. Several chaperones are also induced by these cytokines, which contributes to increasing the efficacy of antigen presentation 40, 43.

## MHC Molecules Are Targeted by Infectious Agents

MHC I molecules are particularly specialized to display peptides of invading or intracellular pathogens to CTLs and initiate activation events that lead to the elimination of infected cells. For survival, pathogens have developed strategies to interfere with the antigen presentation pathway and escape immune surveillance at each stage of this process 44. The EBNA1 antigen from the Epstein–Barr virus (EBV) acts as an inhibitor of proteasomal activity and prevents the generation of immunogenic peptides. The cytomegalovirus (CMV) protein US6 interferes with TAP activity by preventing its binding to ATP, thereby inhibiting peptide translocation and impairing antigen uptake into the ER 45. Tapasin, which associates with TAP and MHC I molecules in the PLC, may also be targeted by the CMV protein US3, resulting in decreased optimization of peptide transport and loading 45. Similarly, adenovirus protein E3‐19K affects the PLC by binding to TAP and preventing its association with tapasin for PLC formation. Alternatively, E3‐19K can also directly interact with MHC I molecules and cause their retention in the ER, blocking antigen presentation 46. Finally, some viruses disrupt MHC I trafficking to the cell surface and cause accelerated endocytosis and lysosomal degradation of MHC I molecules as observed with the HIV protein Nef 47. The multitude of viral proteins devoted to inhibiting MHC I antigen presentation underscores its role in immune surveillance 45, 46, 47, 48. Bacteria have similarly developed tactics to inhibit antigen presentation. The Cif protein is produced by *Pseudomonas aeruginosa* to induce TAP degradation, and intracellular growth by *Salmonella* spp. is reduced to minimize antigenic content in the host cell, among others 49, 50, 51.

## Non‐Classical MHC I Molecules: A Link Between Innate and Adaptive Immunity

Classical MHC I molecules are associated with cellular adaptive immunity. However, non‐classical MHC I molecules, termed MHC Ib, contribute to alternate forms of immune surveillance and immune suppression that support both the innate and adaptive immune response. Although evolutionarily and structurally related, MHC Ib molecules are more limited in their polymorphisms and patterns of expression compared to their classical MHC I counterparts. MHC Ib molecules also include proteins encoded outside the MHC gene locus, possess functions extending beyond peptide presentation, and interact with receptors across both the innate and adaptive immune systems 52, 53, 54, 55, 56. HLA‐E is a well‐characterized MHC Ib molecule with the dual role of regulating both natural killer (NK) and T cells. HLA‐E serves as a critical checkpoint in NK cell‐mediated surveillance that targets tumors and virus‐infected cells, both of which downregulate MHC I molecules to evade immune recognition 53, 57. HLA‐E surface expression is indicative of cells with normal MHC I expression and functional TAP, providing protection against NK cytotoxicity; the NKG2A receptor on NK cells recognizes HLA‐E on target cells to inhibit the lytic process 53, 58. HLA‐E is also recognized by T cells, resulting in the activation of subsets of CD8+ T cells and the adaptive immune response against pathogens such as *Salmonella typhi*, *Mycobacterium tuberculosis*, and the human CMV 52, 53, 56. Another MHC Ib molecule, CD1d, exclusively presents lipid ligands to a population of hybrid NK and T cells termed NKT cells. CD1d‐restricted NKT cells are potent immunomodulators capable of producing Th1 or Th2 cytokines upon activation and acting directly as effector cells with antimicrobial mechanisms 59, 60.

HFE is also a non‐classical MHC Ib molecule, but does not appear to have any antigen‐binding capabilities 61. HFE is ubiquitously expressed and increasing evidence suggests a role in antigen presentation 62 with cross‐talk between HFE and the antigen presentation pathway shown to impair antigen processing and T cell activation 62, 63. Studies have also demonstrated that, although it does not bind peptides, HFE is recognized by T cells and is capable of shaping the T cell repertoire 64, 65 such that CD4/CD8 ratios are imbalanced in HH patients with HFE mutations 66. Furthermore, HFE has also been described as a skin tolerance antigen in pre‐clinical models, with implications in autoimmunity 67. Altogether, these findings suggest a broader immunological role for HFE of growing significance.

## HFE Structure

**Figure 4:**
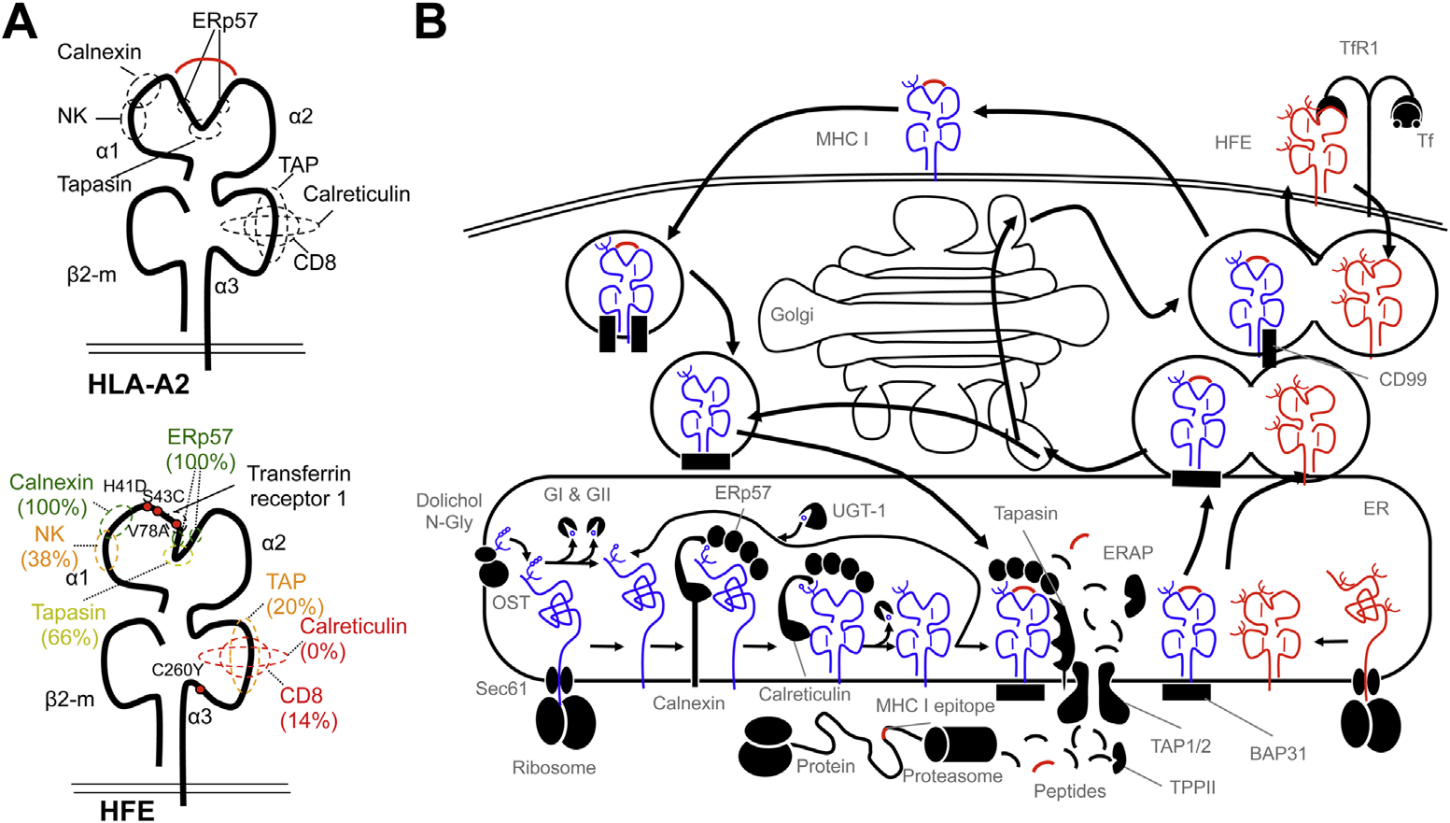
HFE and classical MHC I molecules present homologous structures and are synthesized and folded within the endoplasmic reticulum. A) Structures of HLA‐A2 and HFE and binding sites to antigen presentation chaperones for HLA‐A2, as well as degree of conservation of these sites in HFE. B) Schema depicting the MHC I antigen presentation pathway as well as HFE synthesis, surface transport, and expression.

HFE was originally named HLA‐H due to its homology with the MHC I structure, characterized by a heavy chain comprised of three alpha domains and a transmembrane (TM) domain 9 (Fig. 4A and B). Similar to classical MHC I molecules, HFE requires the binding of the light chain β2‐microglobulin (β2‐m) subunit to its α3 domain for appropriate surface expression 68, 69. The gene encoding HFE is situated on chromosome 6p21.3, close to the HLA‐A locus. Early analysis of the DNA sequence had described HFE as a new MHC I‐like molecule sharing structural homology with MHC I molecules 9. However, HFE was later shown to not present peptides due to a narrow peptide‐binding cleft 18.

Many HFE mutants have been exploited to better characterize the interaction of HFE and TfR1. The most prevalent mutations identified within patients and associated with HH are HFE_C282Y_ and HFE_H63D_ 9, 18. The conversion of a cysteine (C) to a tyrosine (T) in HFE_C282Y_ prevents formation of a disulfide bond located in the α3 domain of HFE, critical for β2‐m binding and protein stability 18, 70, 71. Without β2‐m, the heavy chain of HFE is unable to fold properly or undergo posttranslational processing, and is targeted for degradation 72. Therefore, HFE_C282Y_ prevents extracellular expression of HFE and subsequent interactions with the TfR1, resulting in an increase in cellular iron uptake without HFE interference at the cell surface 18, 70.

The H63D mutation is situated in the α1 domain of HFE and does not affect binding with β2‐m, thereby leaving HFE surface expression unaltered. HFE_H63D_ can still associate with TfR1, however, the substitution of a histidine (H) for an aspartic acid (D) modulates TfR1 affinity for Tf. Whereas wild‐type HFE reduces the affinity of TfR1 for Tf to downregulate iron uptake, HFE_H63D_ was shown to also decrease the TfR1‐Tf interaction though to a lesser degree 18.

## Significance of HFE Splice Variant Expression

HFE is expressed ubiquitously but its expression levels vary greatly from one tissue to another 9, 73. In addition, the expression of different HFE splice variants in the body and their restricted expression patterns suggest their functions may differ based on the tissue type 74, 75. Several splice variants of HFE result in isoforms 75, 76 that may lack one or all of the extracellular (α1‐α3) or transmembrane domains. Quantification of HFE mRNA expression revealed that the liver comprised the highest levels of full‐length HFE, but had the lowest transcript levels of alternative HFE splice variants, emphasizing the importance of HFE_WT_ in the liver for iron metabolism 75. In comparison, the duodenum contained high levels for certain alternative HFE transcripts. Specifically, an HFE isoform containing intron‐4 produces a soluble HFE (sHFE) protein devoid of the transmembrane domain and cytoplasmic tail, and has been detected in transfected cell lines. Furthermore, a sHFE protein complexed with Tf and soluble Tf‐receptor was reported in the serum of healthy individuals 77. A putative role for sHFE was described in regulating systemic iron metabolism, which may have agonist/antagonist effects on HFE_WT_ and implications in responding to iron disorders such as HH 75. More recently, sHFE was shown to control dietary iron absorption in the duodenum through regulation of hephaestin, a membrane‐bound ferroxidase 78. Although the physiological significance of other HFE isoforms has not yet been fully determined, the outcome of these variants may also contribute to the immunological role of HFE.

## HFE Functions in the Immune System

### HFE and NK cells

Non‐classical MHC I (class Ib) molecules such as HLA‐E, ‐F, and ‐G have been shown to bind to NK cells with effects on immunoregulation, autoimmunity, and immune tolerance during pregnancy. NK cells are generally involved in the innate immune response, as they do not require antigen‐specific recognition to kill infected cells 79. NK cells specifically kill cells that lose their surface expression of MHC I 80, a strategy employed to target tumor cells and infected cells 81, 82. An infected cell will be recognized by CD8^+^ T cells through antigen‐specific MHC I molecules, or by NK cells detecting absent or decreased expression of MHC I. Importantly, HFE is not recognized by NK cells 73 in contrast to other MHC Ib molecules such as HLA‐E. Furthermore, the expression of HFE does not alter the reactivity of NK cells 73 nor does it elicit an NK cell response 83. To our knowledge, anomalies in NK populations have not been reported in HH or in other iron overload syndromes.

### HFE and NKT cells

NKT cells are a subset of T lymphocytes that express surface receptors that are characteristic of both T and NK cell lineages 84, 85. NKT cells express a TCR, but unlike conventional T cells, react with lipid or glycolipid antigens presented by the MHC class I‐related glycoprotein CD1d. Most NKT cells, referred to as type I or invariant NKT (iNKT) cells, are defined by their expression of an invariant TCR with particular TCR‐Vβ chains 85. The activation of iNKT cells has been characterized by their ability to recognize α‐galactosylceramide (α‐GalCer), the prototypic CD1d‐restricted glycosphingolipid antigen, which has potent immunoregulatory potential and was the focus of several cancer studies and trials 86, 87. In addition, NKT cells are also activated by exogenous microbial antigens as well as endogenous cellular and tumor‐derived lipid‐based antigens 88.

Recently, a study comparing HH patients and control individuals reported reduced numbers of circulating iNKT in HH patients 89. Untreated patients had more prominent defects in iNKT cells that were reflected in levels of serum ferritin and Tf saturation. These results indicated that iron overload is associated with these reduced numbers of iNKT cells and suggest that HFE may affect the iNKT pool, either directly or indirectly, and potentially act through effects on iron metabolism and iNKT cell homeostasis.

### The link between HFE, CD8^+^ T lymphocytes, and antigen presentation by MHC I

The relationship between HH and modulation of the immune response is strongly suggested by the abundance of immune defects identified in HH patients 90, 91, 92, 93. Both phenotypic and functional abnormalities in the CD8^+^ T cell pool have been associated with HH. Iron can directly affect the phenotype of immune cells, and was shown in vitro to inhibit the surface expression of adhesion molecule CD2 and co‐receptor CD4 on T lymphocytes 94. However, more severe presentations of HH are associated with lower numbers of both circulating and hepatic CD8^+^ T cells, affecting CD4/CD8 ratios 6, and indicating that CD8^+^ T cell numbers may affect intestinal iron absorption levels. The decrease in CD8^+^ T lymphocytes in HH patients appears to be related to defects in the subpopulation that cannot differentiate into CD8^+^ effector memory T cells 95.

Alternatively, other studies have documented an increased presence of some CD8^+^ T cell subsets in HH, such as regulatory CD8^+^CD28^−^ T cells, which coincided with a decrease in CD8^+^CD28^+^ T cells and diminished cytotoxic activity of CTLs 91. In addition, defects in T cell phosphorylation activity were reported with CD8‐associated p56Lck kinase, which is critical for signaling and activation of T cells, and has demonstrated significantly reduced activity in HH patients compared to control individuals 96, 97. Furthermore, the cytokine profiles in HH patients showed a significant increase in levels of IL‐10 and IL‐4 produced by the CD8^+^ T cell subset Tc2, and may encourage Th2 polarization in certain contexts 98.

The findings of all these studies present a strong and undeniable link between the immunity involving CD8^+^ T lymphocytes and HH with associated iron overload. A potential immunological function for HFE has been further implicated with the discovery of HFE and its striking homology with MHC I (Fig. 4A and B). Studies have demonstrated that mutated HFE has a direct impact on MHC I molecules and is associated with abnormal MHC I assembly and expression (Fig. 4C). Peripheral blood mononuclear cells (PBMCs) from HH patients carrying the HFE_C282Y_ mutation were reported to have lower levels of MHC I expression due to an increased rate of MHC I endocytosis. This rapid turnover is caused by accelerated antigenic loading and premature MHC/peptide dissociation that coincides with greater expression of β2‐m‐unbound MHC I heavy chains at the cell surface 63. Further study revealed that misfolded HFE_C282Y_ protein triggers the unfolded protein response (UPR), a mechanism that impacts intracellular trafficking, and gives rise to MHC I anomalies in HFE_C282Y_ cells 99, including reduced cell surface expression. Importantly, despite its inability to present peptides, HFE can be recognized by a TCRαβ of mouse CD8^+^ T cells, particularly those expressing the variable AV6.1 and AV6.6 gene segments 65, further reinforcing a functional link between HFE and antigen presentation by MHC I. These reports have prompted investigations into the role of HFE on CD8^+^ T lymphocyte activation. One study evaluated how the presence of wild‐type and mutated HFE molecules affected the ability of MHC I molecules, specifically HLA‐A2, to present selected antigens and subsequently activate CD8^+^ T lymphocytes 62. Wild‐type HFE, but not HFE_C282Y_, inhibited the secretion of T cell cytokines and the expression of lymphocyte activation markers, demonstrating the functional impact of HFE on CD8^+^ T lymphocytes. The inhibition of CD8^+^ T lymphocyte activation involved the α1–2 domains of wild‐type HFE and was independent of MHC I expression level, β2‐m competition, HFE‐TfR1 interaction, or epitope origin and affinity 62. Considering its ubiquitous expression, these data suggest a new role for wild‐type HFE in altering CD8^+^ T lymphocyte reactivity, which could modulate antigen immunogenicity.

Further support for an HFE role as a negative regulator of CD8^+^ T lymphocyte activation was demonstrated in another study revealing that HFE has an impact on the expression of genes associated with the differentiation, maturation and activation of CD8^+^ T lymphocytes, both in HH patients and in *Hfe*‐deficient mice 100. In particular, HH patients had differential expression patterns for genes involved in the differentiation and maturation of CD8^+^ T memory cells, thereby affecting the homeostatic equilibrium of these cells. The authors proposed that the “low CD8 phenotype” in HH may be the result of a homeostatic equilibrium of cells constantly triggered to activate and differentiate into more mature effector cells 100.

The most obvious implications of HFE as a negative regulator/inhibitor of MHC I antigen presentation and CD8^+^ lymphocyte activation are related to the immune response during infections, cancer immune surveillance, and autoimmunity. A summary of the impact of HFE expression on iron metabolism and MHC I antigen presentation is presented in Table 1.

**Table 1:** Effect of HFE variants on MHC I antigen processing, presentation, and T cell activation

	HFE				
Phenotype	WT	H63D	C282Y	V100A	References
Binds TfR1?	Yes	Yes	No	No	14, 18
Alters hepcidin expression?	Yes	⇓	⇓⇓	NA	133, 134, 135, 136
Affects iron uptake?	⇓⇓	⇑	⇑⇑	⇑⇑	137, 138
Expressed at cell surface?	Yes	Yes	No	Yes	139
Causes ER stress/UPR?	No	Yes	Yes	NA	62, 140, 141
Is unstable degraded?	No	No	Yes	No	140
Alters MHC I chaperone mRNA levels?	No	No	⇑	No	62, 142
Alters MHC I chaperone protein levels?	No	No	No	No	62
Affects MHC I antigen presentation?	⇓⇓	⇓	No	⇓⇓	62
gp100_209–217 _ (melanoma)	⇓⇓	⇓	No	⇓⇓	62
MART‐1_26–35 _ (melanoma)	⇓⇓	⇓	No	NA	62
M1_58–66 _ (influenza)	⇓⇓	⇓	No	⇓⇓	62
Affects high affinity epitopes?	⇓⇓	⇓	No	NA	62
Alters surface MHC I expression?	⇓	No	No	No	62, 63
Binds β2‐m?	Yes	Yes	No	Yes	62
Affects minigene?	⇓⇓	⇓	No	NA	62
Affects pulsed peptides?	No	No	No	No	62
α1‐2 domain homologous to HFE_WT_?	Yes	No	Yes	No	62
Alters proteasome activity?	No	No	No	No	62
Alters ER aminopeptidase activity?	No	No	No	No	62
Alters pan‐cytokine production?	⇓⇓	⇓	No	NA	62
Alters TCR reactivity at surface?	No	No	No	No	62
Alters glycosylation?	No	No	No	No	62
Enriched chaperone binding?	No	No	No	NA	62
Affects CD8 T cell numbers?	No	NA	⇓	NA	6
Affects T lymphocyte activation & signaling?	⇓	NA	No	NA	100
Affects antigen presentation in *trans*?	No	NA	NA	NA	62

Summary of effects observed in investigating the relationship of HFE variants and MHC I antigen presentation in previous studies. Single arrows = moderate effect; double arrows = strong effect.

### Resistance to infection with intracellular pathogens

If mutated HFE_C282Y_ enhances immune responses, then this would likely impact resistance to infections in HH patients. Because most pathogens during infection depend on iron to replicate and survive 101, iron excess in the body is associated with an advantage for pathogen growth. Secondary iron overload can be acquired through multiple blood transfusions used to amend iron imbalances from other diseases or conditions such as thalassaemia which gives rise to anemias or chronic liver disease 6. African siderosis is another iron overload syndrome that affects sub‐Saharan African populations and has limited similarities with HH, but is attributed to dietary consumption of traditional home‐brewed beer that is rich in iron. The excess of iron accumulates significantly in macrophages and other cells of the reticuloendothelial system (RE cells) such that secondary iron overloading is associated with compromised macrophage functions and cellular immunity against pathogens, rendering patients more susceptible to infection 6, 102. In contrast, iron overload from HH is not associated with increased susceptibility to infection or iron loading of phagocytic cells. Of exception are severe infections caused by siderophilic pathogens such as *Vibrius vulnificus* and *Yersinia enterocolitica*, Gram‐negative bacteria that thrive in excess iron 103. *V. vulnificus* infections in HH patients develop into gastroenteritis from raw shellfish, wound infections, and septicaemia; however, it is not known if there is a direct association between mutated HFE_C282Y_ and a higher risk for *Vibrius* infections 104. Interestingly, a recent study by Arezes et al. reported the role of hepcidin in host defense against *V. vulnificus* in hepcidin‐deficient mice 103. Compared to wild‐type mice, hepcidin‐deficient mice were more likely to sustain bacteremia and succumb to fatal infection with *V. vulnificus*. When treated with hepcidin agonists, susceptible mice were rescued from death with the induction of hypoferremia (low iron). This is consistent with the described role for hepcidin in innate immunity which recognizes hepcidin as a defensin‐like antimicrobial peptide, responding to iron overload and inflammation, binding to ferroportin, and causing downstream effects that restrict levels of iron for invading pathogens 11, 103. The results by Arezes et al. showed that hepcidin‐induced hypoferremia was a defense mechanism against pathogens dependent upon iron and revealed hepcidin agonists as potential therapy to improve infection outcome for patients with HH or thalassemia 103.

In general, the impaired iron retention in macrophages from HFE mutation results in an iron deficiency that can attenuate the survival of intracellular pathogens such as *S. typhi*, *M. tuberculosis*, and *Chlamydia pneumoniae*, which depend on high intracellular iron concentrations to multiply in their host cell 6, 105, 106. An increased release of iron with mutated HFE ensures a low intracellular concentration of iron in macrophages and RE cells, creating an inhospitable environment for intracellular pathogens. For facultative intracellular pathogens, a decrease in intracellular iron levels forces replication outside of the cell, exposing the pathogen for rapid clearance by the immune system. More recently, the presence of HFE_C282Y_ was also shown to increase MHC I antigen presentation compared to HFE_WT_ 62. These observations support the hypothesis that HH patients may carry a selective advantage for resistance against infections. Moreover, the prevalence of the HFE_C282Y_ mutation in European populations suggests an evolutionary selection driven by centuries of past pandemics and dietary changes that reflect low availability of iron‐rich foods 107, 108.

### Autoimmunity

From a different perspective, enhanced immune responses by mutated HFE_C282Y_ may favor the appearance of autoimmunity. Various reports have described autoimmune conditions in association with hemochromatosis. In particular a higher prevalence of the HFE_C282Y_ mutation was observed among cases of multiple sclerosis (MS) and was present among MS patients that had an accelerated onset of the disease and more severe MS symptoms 109, 110, 111. Although a direct association has not been established between HFE mutations and MS susceptibility or clinical outcome 109, a recent retrospective study on patients who were homozygous for HFE_C282Y_ concluded that autoimmune conditions were common among individuals with hemochromatosis 15. Expression of the HFE_C282Y_ mutation could increase the self‐reactivity of CD8^+^ T cells that cross the blood‐brain barrier, via increased MHC I antigenic presentation. The HFE_C282Y_ mutation may result in an increased presentation of auto‐antigens related to MS beyond a recognition threshold causing the onset and progression of the disease, unlike HFE_WT_ which could inhibit presentation and maintain immunosuppression 109, 111.

### Cancer immunosurveillance

The implications of HFE mutations in cancer development and progression have been extensively investigated since iron is essential for cell proliferation and is in higher demand in cancer metabolism. To date, no study has established a selective mutation from wild‐type to HFE_C282Y_ or HFE_H63D_ within tumors that would enhance cancer progression 112 and there are contrasting reports on HFE mutations and cancer risk. However, several studies have shown an increased prevalence of HFE_C282Y_ and HFE_H63D_ in tumors, with positive correlations between the presence of the HFE_C282Y_ mutation (heterozygous and homozygous) and the development of cancers such as breast, ovarian, colorectal, and hepatocellular carcinoma 112, 113, 114, 115, 116, 117, 118, 119, 120, 121, 122. With recent evidence highlighting a potential immunological role for HFE in MHC I‐peptide presentation and activation of CD8^+^ T cells, it is plausible that HFE may impact the immune surveillance of tumors 62, 63. Tumor survival is dependent upon evading recognition by the immune system, and often involves deregulating the antigen processing machinery and reducing tumor antigen expression 123. Interestingly, HFE_WT_ rather than HFE_C282Y_, was shown to inhibit MHC I presentation and T cell activation 62. Further investigation showed that HFE expression is higher in tumor cells than in normal human tissues, and is reduced in tumor cells when they are exposed to activated T lymphocytes and soluble mediators, TNF and IFN‐γ 124. In this context, levels of HFE expression in tumor cells may be relevant for reducing tumor immunogenicity and T cell recognition. These results propose a possible balance between pro‐ and anti‐tumor effects that are produced from downregulated HFE expression. While tumor cells may benefit from reduced HFE expression that may increase iron intake (pro‐tumor), the immune system may target tumors by producing cytokines to also reduce tumor HFE expression in order to increase MHC I antigen presentation and facilitate tumor clearance (anti‐tumor) 124.

## Beyond HH: Perspectives on the Immunological Role of HFE

Although HFE cannot present antigens, it actively participates in the MHC I pathway and CD8^+^ T cell activation, revealing an immunological role as a negative regulator of MHC I antigen presentation. HFE association with T cells was also previously highlighted with animal studies that demonstrated that iron overload is more prominent in RAG1 mice deficient in lymphocytes and in HFE‐deficient mice on a RAG1 background 125, 126. Overall, HFE reveals the close relationship between iron metabolism and immunity, and appears to act as a mediator between both processes. HFE_C282Y_ has been associated with the UPR, a cellular stress response affecting the MHC I pathway, which may provide new clues that link UPR signaling pathways and HH pathophysiology 127.

The expression of HFE appears to vary based on immune mediators present in the immediate inflammatory microenvironment 124. Specifically, HFE expression may interfere with an effective anti‐tumor response, in which tumors expressing HFE are exposed to activated T cell‐secreted cytokines that decrease tumor HFE expression. The downregulation of HFE may, in turn, promote MHC I presentation of tumor antigens which are recognized by antigen‐specific cytotoxic CD8^+^ T cells leading to tumor lysis and eradication. These studies may highlight possible mechanisms that involve HFE in the anti‐tumor immune response. In addition, further studies in the expression of HFE variants in different tissues, such as sHFE may further elucidate specific roles of HFE. The α1‐2 domains of HFE_WT_ are responsible for the inhibition of MHC I presentation 62, however, the expression of different splice variants that exclude these domains warrant further study to investigate their immunosuppressive activity.

The immunosuppressive activity of HFE on MHC I antigen presentation may highlight other roles as a mediator in maintaining homeostasis in human tissues. In particular, certain organs have an immune privileged status that is maintained by immune‐suppressive mechanisms and the presence of physical barriers to limit immune infiltration. Some studies suggest that the liver is one of these immune privileged bodies 128, 129, with evidence showing that HFE is most strongly expressed in the liver 9, 73, 130. The high expression of HFE in the liver could suppress MHC I recognition and limit the reactivity of infiltrating CD8^+^ T cells, thereby preventing immune‐derived damage to a central organ of the body. Alternatively, the low levels of HFE detected in the brain 9, 131 suggest that this immune privileged organ may not require HFE protection due to the presence of the blood‐brain barrier which physically restricts access to immune cells 132. This explanation could also be applied to the testicles, which are known to display similarly low levels of HFE and possess a blood‐testis barrier that limits immune infiltration.

## Conclusions

HFE has been described primarily for its role in iron metabolism, however, defining an immunological role for HFE has been of great interest since discovering its remarkable structural homology with MHC I molecules. Recent studies show that, similar to other non‐classical MHC Ib molecules, HFE demonstrates immune activities that bridge innate and adaptive immunity. The immunological abnormalities of HH patients have drawn attention to HFE involvement in CD8^+^ T cell reactivity, and animal studies have highlighted how iron overload is more pronounced in lymphocyte‐deficient mice. In addition, HFE_WT_ has now been shown to inhibit antigen presentation via the MHC I pathway with immunosuppressive effects on CD8 T cells. HFE expression levels may be dependent upon the presence of immune mediators or an inflammatory microenvironment capable of regulating MHC I presentation and of driving the immune response for clearance. While the direct impact of HFE mutations is evident for iron overload, the direct immunological role of these mutations or of HFE isoforms, such as sHFE, is less clear, particularly in the context of immune defects observed in HH patients. HFE mutations, the resulting iron imbalance, or both events may predispose HH patients or modify their response in the development of cancer, autoimmune diseases, and adaptive immunity to pathogens. Further investigation is necessary to characterize the dual roles of HFE and how both the immune system and iron metabolism regulate each other.

## Conflict of Interest

None declared.
